# Types of leisure time physical activities (LTPA) of community-dwelling persons who have been screened positive for dementia

**DOI:** 10.1186/s12877-021-02201-1

**Published:** 2021-04-23

**Authors:** Britta Müller, Peter Kropp, Maria Isabel Cardona, Bernhard Michalowsky, Nanja van den Berg, Stefan Teipel, Wolfgang Hoffmann, Jochen René Thyrian

**Affiliations:** 1 Institute of Medical Psychology and Medical Sociology, University Medicine Rostock, Gehlsheimer Str. 20 Rostock, 18147 Rostock, Germany; 2 German Center for Neurodegenerative Diseases (DZNE), Greifswald, Germany; 3 Institute for Community Medicine, University Medicine Greifswald, Greifswald, Germany; 4 German Center for Neurodegenerative Diseases (DZNE), Rostock, Germany; 5 Department of Psychosomatic and Psychotherapeutic Medicine, University Medicine Rostock, Rostock, Germany

**Keywords:** Dementia, Physical activity, Rural, Aging

## Abstract

**Background:**

To (a) describe the pattern of leisure time physical activities (LTPA) in community-dwelling persons who have been screened positive for dementia and (b) determine the health-related and sociodemographic factors associated with LTPA.

**Methods:**

Data of the general practitioner-based, randomized, controlled intervention trial, DelpHi-MV (Dementia: life- and person-centered help in Mecklenburg-Western Pomerania) were used. Patients aged 70 years or older, who lived at home and had a DemTect< 9 were informed about the study by their General practitioners and invited to participate. Data from 436 participants with complete baseline data were used. Standardized, computer-assisted assessments were made during face-to-face interviews at the participants’ homes.

**Results:**

Two hundred thirty-eight patients (54.6%) carried out LTPA (men 58.4%, women 51.8%). Physically active patients mentioned one to two different activities; diversity of LTPA was higher for men than for women. The most-frequently mentioned types of activity were gardening (35.3%), cycling (24.1%) and mobility training (12.4%); there was only a statistically significant difference between men and women in cycling, χ^2^(1) = 21.47, *p* < .001. The odds of LTPA increased with increasing quality of life (*OR* = 2.41), lower impairments in activities of daily living (*OR* = 0.85), and living in a rural environment (*OR* = 2.02).

**Conclusions:**

Our findings suggest that people who have been screened positive for dementia living in a rural area are more likely to be active than people living in an urban area. Following studies should investigate whether this difference has an effect on the progression of dementia.

**Trial registration:**

ClinicalTrial.gov Identifier NCT01401582.

**Supplementary Information:**

The online version contains supplementary material available at 10.1186/s12877-021-02201-1.

## Background

There is broad evidence for the beneficial effects of physical activity in patients with dementia. Physically active persons with cognitive impairment have a lower risk for mortality than physically inactive persons with dementia [1]. Physical activity may lead to improvements in gait speed [2] endurance [3, 4], balance [3, 4], and muscle strength [3, 4]. It may reduce the risk of falls [5], increase daily living activities [6, 7] attenuate depressive symptoms [6, 8], and slow cognitive decline [2, 4, 6, 7, 9]. Additionally, physical activity among persons with dementia contributes to maintaining self-hood [8] and enhances the quality of life [10].

However, little is known about the level of physical activity in people with cognitive impairment living at home and the factors associated. Compared to healthy older adults, dementia patients spend significantly more time awake in a sedentary state and significantly less time in light-to-moderate and moderate-to-strenuous activities. This may have clinically important consequences considering the observation in previous prospective studies that sedentary behaviour independently predicts overall mortality and morbidity [11–13]. There is evidence from a systematic review that among community-dwelling adults with dementia, activities of daily living (ADL), gait speed, nutrition and quality of life were positively associated with physical activity, whereas age and cognitive status were not related to physical activity [14]. The role of caregivers in dementia patient’s physical activity participation is pointed out by two studies. In their systematic review van Alphen, Hortobagyi [15] showed that difficulties with guidance and organization of physical activities by caregivers is a prominent barrier to physical activity. Farina, Williams [16] reported, based on a qualitative study, that physical activity of persons with dementia can be promoted by supporting caregivers in general and by facilitating the caregiver’s own physical activity. However, previous findings were mostly based on small samples and did not account for sex-specific effects. In addition, other variables that might contribute to differences, like i.e. urban vs. rural environment, have not been studied.

There is a clear public health need to measure physical activity behavior and to identify factors associated with dementia in older adults. This may result in the development of effective interventions for promoting regular physical activity among this population [17].

Thus, the aim of the present analysis is to (a) describe the pattern of leisure time physical activities (LTPA) in community-dwelling persons who have been screened positive for dementia and (b) determine which health-related and sociodemographic factors are associated with LTPA. These findings will be relevant for a better understanding of factors that contribute to or result from LTPA in dementia and may serve as proxies for treatment monitoring or as targets for interventions.

## Methods

### Study design

The analyses are based on data of the general practitioner (GP)-based, randomized, controlled intervention trial, DelpHi-MV (Dementia: life- and person-centered help in Mecklenburg-Western Pomerania). The DelpHi-intervention aims to provide “optimum care” by integrating multi-professional and multimodal strategies to individualize and optimize treatment of dementia within the framework of the established healthcare and social service system. Participants randomised to the intervention group received improved integrative and collaborative care conducted by Dementia Care Managers (DCM) – nurses with dementia-specific training – at the people’s home. The intervention has been shown to be effective and results of this trial on disease–oriented outcomes have been published [18, 19]. The control group received “care as usual” [20]. The details of this trial are described elsewhere [21], the ClinicalTrial.gov identifier is NCT01401582. Patients aged 70 years or older and living at home were systematically screened for dementia by their treating GPs during routine visits using DemTect [22]. This personal interview-based instrument is widely used for dementia screening in GP practices in Germany [23]. Patients who met the inclusion criteria for DelpHi-MV (DemTect< 9) were informed about the study by their GPs, invited to participate and asked to provide written informed consent. Patients who included in the study were contacted by their designated DCM to arrange two to three personal visits to carry out the computer-assisted comprehensive standardized baseline assessments as face-to-face interviews at the participant’s home. When the patient was unable to give written informed consent, his or her legal representative was asked to sign the consent form on his or her behalf (as approved by the Ethics Committee of the Chamber of Physicians of Mecklenburg-Western Pomerania, registry number BB 20/11). The study physicians received allowances for performing the screening test (10€ per patient) and study enrollment (100€ per patient). Study enrollment into the main study started on January 1, 2012 and ended on December 31, 2014. 

### Study population

A total of 6838 patients were screened for dementia in 125 participating GP practices. Of these, 1167 patients (17.1%) were eligible for the DelpHi-MV trial, and 634 patients (54.4%) agreed to participate in the study. One-hundred-eighteen patients were not enrolled in of the study before the baseline assessment due to withdrawal of informed consent (*n* = 85), death (*n* = 19), relocation (*n* = 5) or other (*n* = 9). There were no statistically-significant differences in the DemTect score, age or sex between the patients who were included and those who dropped out. A detailed description of the study population is provided according to the CONSORT reporting standard in Thyrian, Eichler [24] and Thyrian, Hertel [18].

Finally, 516 patients at 94 GP practices completed the baseline assessment of the study. Of those 516 patients, 80 (15.5%) were excluded from the present analysis due to missing values. The reasons for this were dropping out during the baseline assessment (*n* = 17), too impaired to follow test instructions (*n* = 56) and the response “don’t know” (*n *= 7)*.* Missing data were more frequent among patients with more severe cognitive impairment (DemTect-score, mean (*SD*): included patients 6.13 (1.85), excluded patients 4.33 (2.56), *t* (514) = 6.02, *p* < .001). Thus, 436 participants with complete baseline data on physical activity, health and social context were entered into the statistical analysis.

### Procedures and instruments

Data about LTPA were obtained based on a multiple response question. The participants were asked to indicate whether they carried out one or more of the following seven activities: mobility training in sports groups for senior citizens, cycling, gardening, going for a walk, swimming, bowling, and dancing. These items were formulated dichotomously (yes/no). Data regarding frequency, duration or time frame, in which the activities took place, were not collected*.* Activities explicitly asked were chosen based on the consensus of experts in the field. They were considered to be the most prevalent and relevant for this population and for this area. The use of more comprehensive, validated physical activity questionnaires was waived to keep the burden of data assessment as low as possible for the sample. Additionally, participants were asked to indicate further activities in two open questions (“other sports” and “other”). The cognitive impairment of our sample limits information-related recall [25]. This raises the question of the appropriateness of using self-report physical activity questionnaires in people with dementia. In addition, it must be acknowledged that autobiographical memory and episodic memory deficits are common in people with dementia resulting in less detail and more overgeneralization [26, 27]. Therefore, successful use of self-report questionnaires for people with dementia requires: 1) Shortened questionnaire length to minimise burden on the person with dementia; 2) Greater focus on light physical activities rather than more intense activities since people with dementia remain relatively sedentary; 3) Use of prompts, cued recall or recognition (rather than spontaneous recall); 4) Use of more general questions about physical habits, rather than the recall of specific activities based on their duration and timing [28]. The multiple response question we used in our study meets three of these four requirements. It is short (nine responses), seven of nine responses are based on cued recalls and contain general items.

Participants were categorized as physically active (more than low) if they named at least one activity other than “going for a walk”. To go for a walk (German: Spazierengehen) implies a great variety of activities. This regards the distance, speed, change from stop and go, and the duration of breaks. In current German the use of “to go for a walk” subsummizes brisk walking, strolling, windowshopping, or going into the fresh air. To avoid forcing participants to differentiate common terms and to minimize the burden on the participants caused by a high number of activities, we did not make any further differentiations of walking in the question. Due to the fact that not all of these activities are in line with physical activity, we did not take “going for a walk” into account as physical activity (more than low). Participants who did not name any of the activities (or only named “going for a walk”) were defined as physically low/ no active. Years of education were calculated on the basis of the highest level of educational and vocational qualifications achieved. We determined the number of years required to achieve educational and vocational qualifications, and the sum of educational and vocational qualification years then yielded the total years of education [29]. Social support was measured using the short form (22 items) of the Social Support Questionnaire (F-SozU), which measures the expected social support from one’s social environment [30]. We used the mean item score, which has a range from 1 to 5, with higher scores indicating better social support. To measure cognitive impairments, we used the German version of the Mini-Mental State Examination (MMSE) [31]. Impairments in activities of daily living were measured with the Bayer Activities of Daily Living Scale (B-ADL), an instrument that was developed for patients with a decline in cognitive performance [32]. The mean item score ranges from 1 to 10, with high values indicating strong impairments. We used the short form of the Geriatric Depression Scale (GDS), which encompasses 15 dichotomous items and has scores ranging from 0 to 15 [33]. Quality of life was assessed using the Quality of Life in Alzheimer’s Disease (QoL-AD) scale, which comprises 13 items rated on a 4-point scale, yielding a total score ranging from 13 to 52 [34], with a higher score indicating better self-rated quality of life.

### Statistical analysis

The variables describing the sample were analyzed for the total sample and for women and men separately. To test for differences between subgroups, we used Fisher’s exact test, Welch’s *t*-test and Pearson’s chi-squared test. Multiple comparisons were adjusted using the Holm-Bonferroni procedure [35]. To identify the factors associated with physical activity (more than low), we first calculated univariate binary logistic regressions that included health parameters (i.e., quality of life, depression, functional impairment, cognitive impairment, incontinence and pain), social parameters (i.e.*,* living in partnership, perceived social support, living environment, living alone, support of informal caregiver) and education. On the basis of the univariate models, multivariate binary logistic regression models were calculated. The requirements of no multicollinearity and linear relationship between independent variables and the logit of the dependent variable were fulfilled. GP was considered as random effects cluster variable using library lmerTest in R. All other statistical analyses were performed using IBM SPSS Statistics 23.

## Results

### Sociodemographic and health-related characteristics

The sociodemographic and health-related characteristics of the sample are presented in Table 1. The mean FSozU score of our sample was 4.00 and corresponds to that of a general population sample [30].

**Table 1:** Sociodemographic and health-related characteristics of the study sample

Variable		Total Sample	Men	Women	*p* Value
		*n* = 436	*n* = 185	*n* = 251	
Age, mean (*SD*)		80.1 (5.34)	78.52 (5.01)	81.34 (5.27)	< .001^b^
Years of education, mean (*SD*)		9.55 (2.15)	10.25 (2.58)	9.02 (1.59)	< .001^b^
Marital status	Unmarried, *n* (%)	24 (5.5)	12 (6.5)	12 (4.8)	< .001^a^
	Married, *n* (%)	193 (44.3)	124 (67.0)	69 (27.5)	
	Divorced, *n* (%)	29 (6.7)	10 (5.4)	19 (7.6)	
	Widowed, *n* (%)	190 (43.6)	39 (21.1)	151 (60.2)	
Living with partner (no), *n* (%)		214 (49.1)	45 (24.3)	169 (67.3)	< .001^a^
Living alone (yes), *n* (%)		216 (49.5)	61 (33.0)	155 (61.8)	< .001^a^
Living environment (urban), *n* (%)		291 (66.7)	118 (63.8)	173 (68.9)	.260^a^
Support of informal caregiver (no), *n* (%)		136 (31.3)	56 (30.3)	80 (32.1)	.680^a^
Support of informal caregiver (yes), *n* (%)	Spouse/ partner, *n* (%)	135 (45.5)	94 (72.9)	41 (24.4)	< .001^a^
	Daughter/ son, *n* (%)	110 (36.9)	24 (18.6)	86 (50.9)	
	Daughter-/ son-in-law, *n* (%)	15 (5.0)	3 (2.3)	12 (7.1)	
	Granddaughter/ -son, *n* (%)	11 (3.7)	1 (0.8)	10 (5.9)	
	Others, *n* (%)	27 (9.1)	7 (5.4)	20 (11.8)	
Perceived social support (F-SozU), mean (*SD*)		4.00 (0.67)	4.01 (0.74)	3.99 (0.61)	.810^b^
Cognitive impairment (MMSE)	Score, mean (*SD*)	22.72 (4.80)	23.23 (4.88)	22.33 (4.71)	.053^b^
	None (score, 27–30), *n* (%)	105 (24.1)	55 (29.7)	50 (20.0)	.130^a^
	Mild (score, 20–26), *n* (%)	231 (53.1)	94 (50.8)	137 (54.8)	
	Moderate (score, 10–19), *n* (%)	93 (21.4)	34 (18.4)	59 (23.6)	
	Severe (score, 0–9), *n* (%)	6 (1.4)	2 (1.1)	4 (1.6)	
Depressive symptoms (GDS > 5) (yes), *n* (%)		67 (15.4)	24 (13.0)	43 (17.2)	.236^a^
Incontinence (yes), *n* (%)		164 (37.7)	47 (25.4)	117 (46.8)	< .001^a^
Pain, last 4 weeks (yes), *n* (%)		251 (57.6)	104 (56.2)	147 (58.6)	.624^a^
Functional impairment (B-ADL), score, mean (*SD*)		3.50 (2.36)	3.24 (2.27)	3.68 (2.41)	.051^b^
Antidepressive drug treatment (yes), *n* (%)		63 (14.4)	12 (6.5)	51 (20.3)	< .001
Quality of life (QoL-AD), score, mean (*SD*)		2.78 (0.36)	2.80 (0.35)	2.77 (0.37)	.290^b^

*F-SozU* Social Support Questionnaire, mean score 1–5, higher score indicates better social support, *MMSE* Mini-Mental State Examination, range 0–30, higher score indicates better cognitive functioning, *GDS* Geriatric Depression Scale, sum score 0–15, score > 5 indicates depression, *B-ADL* Bayer Activities of Daily Living Scale, range 0–10, lower score indicates better performance, *QoL-AD* Quality of Life in Alzheimer’s Disease scale, mean sum score 1–4, higher score indicates better quality of life, *SD* Standard Deviation; ^a^Pearson’s chi-squared test; ^b^Welch’s *t*-test

### Proportions of LTPA

Of the participants in our sample, 54.6% (95%-*CI* 49.9–59.3) carried out LTPA (more than low). Of the total sample, 31% pursued one activity, 17% did two activities and 7% did three activities. On average, participants mentioned one to two activities (mean = 1.55; *SD* = 0.70). The diversity of LTPA (more than low) was higher for men (mean = 1.67; *SD* = 0.77) than for women (mean = 1.46; *SD* = 0.62), *t* (236) = 2.22, *p* = .028. The most frequently-mentioned types of activity were as follows: 35.3% gardening, 24.1% cycling, 12.4% mobility training in sports groups for senior citizens, 7.3% dancing, 4.6% swimming, 3.2% other. There was a statistically significant difference between men and women only in cycling, χ^2^(1) = 21.47, *p* < .001 (see Table 2). 76.1% (*n* = 332) of the study participants reported going for a walk (men 74.1%, women 77.7%, χ^2^(1) = 0.78, *p = *.426). Of these, 134 named going for a walk as their only activity. We classified these 134 people as physically low active or inactive patients, in addition to those 64 persons who did not engage in any LTPA. The sociodemographic characteristics of both subgroups are shown in the Additional file 1: Appendix.

**Table 2:** Prevalences of types of LTPA for the total sample and for men and women; in % (95%-*CI*)

Tyes of LTPA	Total Sample *n* = 436	Men *n* = 185	Women *n* = 251	*p* Value (adj)^c^
Gardening	35.3 (30.8-39.8)	40.5 (33.4-47.6)	31.5 (25.8-37.2)	.200^a^
Cycling	24.1 (20.1-28.1)	35.1 (28.2-42.0)	15.9 (11.4-20.4)	< .001^a^
Mobility Training	12.4 (9.3-15.5)	7.6 (3.8-11.4))	15.9 (11.4-20.4)	.054^a^
Dancing	7.3 (4.9-9.8)	6.5 (3.0-10.0)	8.0 (4.7-11.3)	1.00^a^
Swimming	4.6 (2.6-6.6)	7.0 (3.3-10.7)	2.8 (0.8-4.8)	.185^a^
Bowling	1.4 (0.3-2.5)	3.2 (0.7-5.7)	1.6 (0.05-3.1)	.744^b^
Other	3.2 (1.5-4.9)	2.7 (0.4-5.0)	2.8 (0.8-4.8)	1.00^a^

^a^Pearson’s chi-squared test; ^b^Fisher’s exact test; ^c^Adjusted *p*-values based on the Holm-Bonferroni method; *CI* Confidence Interval

### Characteristics of patients according LTPA

There were significant differences between participants with more than low and low/ no LTPA regarding living with partner, *χ*^*2*^_(1, *N* = 436)_ = 13.1, *p =* .004, living environment, *χ*^*2*^_(1, *N* = 436)_ = 10.47, *p =* .012, and depressive symptoms, *χ*^*2*^_(1, *N* = 436)_ = 12.84, *p =* .005. Participants with LTPA (more than low) were younger, *t* (434) = 3.45, *p =* .006, perceived more social support, *t* (434) = − 4.30, *p* < .001, were less impaired in activities of daily living, *t* (434) = 5.46, *p* < .001, and experienced more quality of life, *t* (434) = − 5.76, *p* < .001 (see Table 3).

**Table 3:** Sociodemographic and health-related characteristics of physically active (more than low) and low/ no active patients

Variable	Physically active patients (more than low) *n* = 238	Physically low/ no active patients *n* = 198	*p* Value (adj.)^c^
Age, mean (*SD*)	79.34 (4.93)	81.06 (5.62)	.006^b^
Sex (female), *n* (%)	130 (54.6)	121 (61.1)	.497^a^
Years of education, mean (*SD*)	9.74 (2.18)	9.31 (2.10)	.253^b^
Living with partner (no), *n* (%)	98 (41.2)	116 (58.6)	.004^a^
Living alone (yes), *n* (%)	107 (45.0)	109 (55.1)	.258^a^
Living environment (urban), *n* (%)	143 (60.1)	148 (74.7)	.012^a^
Support of informal caregiver (no), *n* (%)	84 (35.4)	52 (26.4)	.258^a^
Perceived social support (F-SozU), mean (*SD*)	4.12 (0.63)	3.85 (0.68)	< .001^b^
Cognitive impairment (MMSE), score, mean (*SD*)	23.04 (4.62)	22.34 (4.99)	.497^b^
Depressive symptoms (GDS > 5) (yes), *n* (%)	23 (9.7)	44 (22.2)	.005^a^
Incontinence (yes), *n* (%)	82 (34.5)	82 (41.6)	.497^a^
Pain, last 4 weeks (yes), *n* (%)	134 (56.3)	117 (59.1)	.561^a^
Functional impairment (B-ADL), score, mean (*SD*)	2.94 (2.10)	4.15 (2.48)	<.001^b^
Quality of life (QoL-AD), score, mean (*SD*)	2.87 (0.35)	2.67 (0.35)	<.001^b^

*F-SozU* Social Support Questionnaire, mean score 1–5, higher score indicates better social support, *MMSE* Mini-Mental State Examination, range 0–30, higher score indicates better cognitive functioning, *GDS* Geriatric Depression Scale, sum score 0–15, score > 5 indicates depression, *B-ADL* Bayer Activities of Daily Living Scale, range 0–10, lower score indicates better performance, *QoL-AD* Quality of Life in Alzheimer’s Disease scale, mean sum score 1–4, higher score indicates better quality of life, *SD* Standard Deviation; ^a^ Pearson’s chi-squared test; ^b^ Welch’s *t*-test; ^c^ Adjusted *p*-values based on the Holm-Bonferroni method

### Factors associated with LTPA: multivariate analysis

Logistic regression was performed to assess the impact of living with partner, living environment, depressive symptoms, age, perceived social support, functional impairments, and quality of life on the likelihood that persons who have been screened positive for dementia would carry out LTPA (more than low). The overall significance of the model was, *χ*^*2*^_(7, *N* = 434)_ = 74.31, *p <* .001 (Likelihood-Ratio-Test). It explained between 15.7% (Cox and Snell *R*^2^) and 21.0% (Nagelkerke *R*^2^) of the variance in LTPA and correctly classified 67.1% of cases. Only three of the seven independent variables made a unique statistically-significant contribution to the model: functional impairment, (*OR* = .85; 95%-*CI*: 0.77–0.934, *p* = .001), quality of life, (*OR* = 2.41; 95%-*CI*: 1.12–5.19, *p* = .025), and living environment, (*OR* = 2.02; 95%-*CI*: 1.29–3.17, *p* = .002). The effects remained essentially unchanged when considering GP as a random effects cluster variable in a generalized mixed effect regression using the library lmerTest in R. The detailed model is provided in Table 4. As functional impairment increased, the odds of LTPA (more than low) decreased. A one-unit increase in the quality of life score increased the odds of LTPA (more than low) by 2.40. The odds ratio for living environment indicated that persons living in a rural environment were two times more likely to carry out LTPA (more than low) than persons living in a urban environment.

**Table 4:** Factors associated with LTPA (more than low) (yes): Total Sample

Potential Factor	Multivariate Model		
	Adj. *OR*	95% *CI*	*p* Value
Functional impairment (B-ADL), score	0.85	0.77–0.93	.001
Rural environment (urban^a^)	2.02	1.30–3.17	.002
Quality of life (QoL-AD), Score	2.41	1.12–5.19	.025
Age (years)	0.96	0.92–1.00	.053
Living with partner (no^a^)	1.51	0.97–2.34	.065
Perceived social support (F-SozU)	1.37	0.94–1.99	.105
Depressive symptoms (GDS > 5) (yes^a^)	1.35	0.71–2.59	.360

*OR* odds ratio, *CI* Confidence Interval, *F-SozU* Social Support Questionnaire, mean score 1–5, higher score indicates better social support, *GDS* Geriatric Depression Scale, sum score 0–15, score > 5 indicates depression, *B-ADL* Bayer Activities of Daily Living Scale, range 0–10, lower score indicates better performance, *QoL-AD* Quality of Life in Alzheimer’s Disease scale, mean sum score 1–4, higher score indicates better quality of life; ^a^reference

### Pattern of LTPA depending on living environment, functional impairment and quality of life

**Fig. 1:**
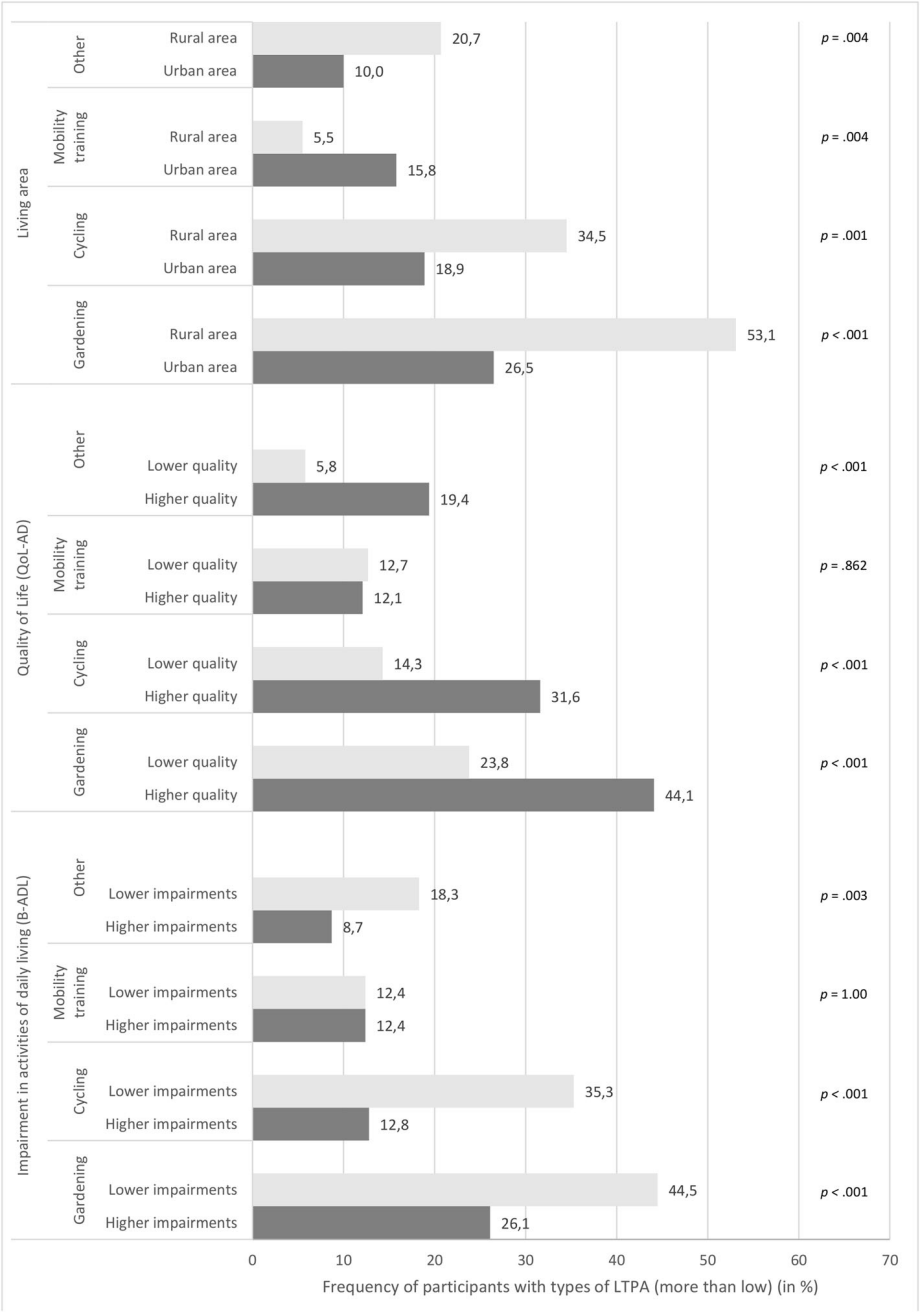
Frequency of participants with types of LTPA depending on living area, quality of life and impairments in activities of daily living (adjusted *p*-values)

Compared to persons with lower impairments in ADL, persons with higher impairments had smaller proportions in gardening, χ^2^(1) = 16.1, *p <* .001, cycling, χ^2^(1) = 30.1, *p <* .001, and other, χ^2^(1) = 8.6, *p =* .003. Persons with lower quality of life participated less than persons with higher quality of life in gardening, χ^2^(1) = 19.3, *p <* .001, cycling, χ^2^(1) = 17.5, *p <* .001, and other, χ^2^(1) = 17.0, *p <* .001. The percentages of participants who did gardening, cycling, and other are higher among rural dwellers than among urban dwellers, gardening, χ^2^(1) = 30.1, *p <* .001, cycling, χ^2^(1) = 12.9, *p <* .001, and other, χ^2^(1) = 9.5, *p =* .002. The proportions of users of mobility training in sport groups were higher in urban than in rural areas, χ^2^(1) = 9.4, *p =* .002 (see Fig. 1).

## Discussion

Based on data of the DelpHi-MV trial we analyzed proportions and pattern of leisure time physical activities (LTPA) in community-dwelling persons who had been screened positive for dementia. Moreover, we analyzed factors associated with LTPA.  In our study, 54.6% of the participants were physically active (more than low). The corresponding percentages in the literature have been reported to range from 32 to 70% [36, 37]. The variability is mainly due to different instruments used to measure both cognitive impairment and physical activity. However, there is evidence that physical activity is lower in cognitively-impaired persons than in persons without cognitive impairments [36–38], and in persons with dementia compared to cognitively-healthy persons [12, 39–41]. We found that the most frequently-mentioned types of activity were gardening (35.3%), cycling (24.1%), and mobility training in sport groups (12.4%). The high prevalence of gardening corresponds to findings from the gerontological literature on persons without dementia [42]. Cycling is also described in the literature as a frequent leisure time activity in older people [43–45]. The comparatively low proportion of participants in our sample who were active in sports groups for senior citizens is likewise consistent with the literature [45–49]. The various findings mentioned above are valid for both European and North American countries. It seems that the preferences of LTPA of people with cognitive impairment are consistent with those of older, cognitively-healthy people.

We could show that three factors were associated with LTPA among persons screened positive for dementia: functional impairment, quality of life, and living environment.

First, the finding that LTPA (more than low) was less likely with increasing functional impairment is consistent with studies that examined older cognitively-unaffected populations [50, 51]. However, we were able to confirm this association in persons with cognitive impairment. Given that we analysed cross-sectional data, we cannot make any conclusions about the causal relationship. There is evidence for both the functional health benefits of LTPA [45, 46] and the limitations of LTPA due to functional impairment [16]. Furthermore, depression may mediate the relationship between LTPA and functional impairment [52, 53]. Our results showed that persons with more functional impairments cycle and garden less often than persons with less functional impairments. We suspect that cycling is, on the one hand, a proxy for higher functional capacity. On the other hand, it is to be expected that functional impairments may reduce the probability of cycling. We assume that functional impairments do not affect implicit bicycling skills as part of the procedural memory in our sample, but rather the orientation and the behaviour as road user. The B-ADL measures complex, instrumental activities (IADLs) (e.g. doing two things at the same time, finding the way in an unfamiliar place, coping with unfamiliar situations) [32] which reflect attention and executive functions (e.g. estimating and anticipating dangerous situations, rule compliance). Additionally, persons with more cognitive impairments may become less confident about their abilities and therefore they skip their favoured activities. 

Second, we found a positive association between likelihood of LTPA (more than low) and quality of life regarding gardening and cycling. Findings regarding changes of quality of life as a result of LTPA interventions in older people with cognitive impairment or subjective cognitive decline are inconsistent [34, 54, 55]. Various studies have reported that horticultural therapy of patients with dementia improved well-being [56–58]. These results can also be transferred to gardening activities of community-dwelling cognitively-impaired persons. Physical acitivity during gardening combined with a sense of achievement can result in an increased sense of self-esteem and relief from stress. With regard to cycling, we assume that it might stabilize the quality of life by maintaining a sense of control, autonomy and resulting in satisfaction by achieving goals. Third, our most important result is the higher probability of LTPA (more than low) in the rural compared to the urban population. In our study rural dwellers live in communities with less than 5000 residents. Urban dwellers live predominantly in small (5000 to less than 20,000 residents) and medium-sized towns (up to 100,000 residents). Rural and urban differences in LTPA among community-dwelling adults with cognitive impairments are not well documented [14]. We could show that the most common domain of LTPA, gardening, is done more often by rural dwellers than urban dwellers. It is evident that gardening is more prominent in rural than in urban conditions. Gardening in urban conditions takes place in allotments, which are further away from the apartments. In old age, when gardening or covering the distance from home togarden become too strenuous due to increasing health limitation, such as cognitive impairments, people often decide to discontinue gardening. In contrast, most of the rural study population lives in single family houses with gardens, some of which used to be farms. When physical and cognitive capacities decrease with age, elderly people in rural areas often decide to reduce gardening but not to discontinue it. Rural gardening may be based on decades of practice. Consequentliy these activities become more and more implicit routines which do not depend on declarative or explicit functions. They may therefore be better preserved in older age. Recent studies report an impact of urbanization on dementia: older adults in urban areas appear to have lower risk of dementia than rural dwellers. The reasons of this association are not well understood. Mediator-variables are discussed, such as education and access to social and health services. We suspect that the differences in dementia risk between rural and urban dwellers would be even greater if LTPA of rural dwellers were at the same low level as in the older urban population.

Our study has several limitations. First, the validity of self-reported LTPA in a sample of patients who have been screened positive for dementia may be lower than in persons without cognitive impairments. To limit this bias, we asked about physical activities regardless of their intensity, duration and frequency. Second, we are aware that classifying people as physically low or no active if they reported going for a walk as their only activity has weaknesses, as the frequency and duration of this activity has not been directly surveyed*.* Third, as we used a cross-sectional study design, we cannot draw conclusions about the causality of the identified associations. Fourth, the generalizability of the findings is limited to community-dwelling persons who have been screened positive for dementia in medium-sized and small towns as well as rural communities in one federal state of Germany. Further studies are needed that replicate our findings for different settings like living situations (care facilities, different states, different countries, areas with different population densities, larger cities etc.).

## Conclusions

54.6% of the participants who have been screened positive for dementia were physically active. The most frequently-mentioned types of activity were gardening (35.3%), cycling (24.1%), and mobility training in sport groups (12.4%). In addition to the expected predictors quality of life and impairments in activities of daily living, the living environment also impacted LTPA. Our results have various implications. First, they suggest that among community-dwelling individuals with cognitive impairments, those living in urban areas would particularly benefit from LTPA promotion. Second, further work is needed to analyse how community-dwelling and cognitively impaired individuals can remain physically active despite increasing functional impairments.

## Supplementary Information

**Additional file 1.**

## Data Availability

The datasets used and analyzed during the current study are available from the corresponding author on reasonable request.

## Abbreviations

DelpHi-MV: Dementia: life- and person-centered help in Mecklenburg-Western Pomerania
GP: General practitioner
LTPA: Leisure time physical activity
