# Supplementary material for: Types of leisure time physical activities (LTPA) of community-dwelling persons who have been screened positive for dementia
Source: BMC Geriatr. 2021 Apr 23;21:270. doi: 10.1186/s12877-021-02201-1 (PMC8063325; doi:10.1186/s12877-021-02201-1)
Supplement: Supplementary file 1 — Additional file 1. [file 12877_2021_2201_MOESM1_ESM.docx]

Appendix. *Sociodemographic and health related characteristics of patients classified as physically low/ no active participants*

| Variables | Participants reported only going for a walk | Participants reported no activity | *p* value |
| --- | --- | --- | --- |
|  | *n*=134 | *n*=64 |  |
| Age, mean (*SD*) | 80.85 (5.66) | 81.66 (5.64) | .452^b^ |
| Sex (female), *n* (%) | 82 (61.2) | 39 (60.9) | .972^a^ |
| Years of education, mean (*SD*) | 9.36 (2.21) | 9.20 (1.84) | .933^b^ |
| Living with partner (no), *n* (%) | 78 (58.2) | 38 (59.4) | 1.00^a^ |
| Living alone (yes), *n* (%) | 80 (59.7) | 29 (45.3) | .067^a^ |
| Living environment (urban), *n* (%) | 106 (79.1) | 42 (65.6) | .054^a^ |
| Support of informal caregiver (no), *n* (%) | 37 (27.8) | 15 (23.4) | .606^a^ |
| Perceived social support (FSozu), mean (*SD*) | 3.87 (.66) | 3.80 (.73) | .707^b^ |
| Cognitive impairment (MMSE), score, mean (*SD*) | 22.50 (5.03) | 21.97 (4.90) | .455^b^ |
| Depressive symptoms (GDS>5) (yes), *n* (%) | 26 (19.4) | 18 (28.1) | .201^a^ |
| Incontinence (yes), *n* (%) | 50 (37.6) | 32 (50.0) | .123^a^ |
| Pain, last 4 weeks (yes), *n* (%) | 79 (59.0) | 38 (59.4) | 1.000^a^ |
| Functional impairment (B-ADL), score, mean (*SD*) | 3.81 (2.31) | 4.90 (2.67) | .007^b^ |
| Quality of life (QoL-AD), score, mean (*SD*) | 2.71 (.35) | 2.60 (.34) | .041^b^ |

FSozu, Social Support Questionnaire, mean score 1-5, higher score indicates better social support; MMSE, Mini-Mental State Examination, range 0-30, higher score indicates better cognitive functioning; GDS, Geriatric Depression Scale, sum score 0-15, score > 5 indicates depression; B-ADL, Bayer Activities of Daily Living Scale, range 0-10, lower score indicates better performance; QoL-AD, Quality of Life in Alzheimer´s Disease Scale, mean sum score 1-4, higher score indicates better quality of life; SD: standard deviation, ^a^ Fisher’s exact test, ^b^ Welch´s *t*-test.
